# Gaming With Stigma: Analysis of Messages About Mental Illnesses in Video Games

**DOI:** 10.2196/12418

**Published:** 2019-05-08

**Authors:** Manuela Ferrari, Sarah V McIlwaine, Gerald Jordan, Jai L Shah, Shalini Lal, Srividya N Iyer

**Affiliations:** 1 Prevention and Early Intervention Program for Psychosis Douglas Mental Health University Institute Montreal, QC Canada; 2 Department of Psychiatry McGill University Montreal, QC Canada; 3 Yale Program for Recovery and Community Health Yale University New Haven, CT United States; 4 Carrefour de l'innovation et de l'évaluation en santé Centre de Recherche du Centre Hospitalier de l'Université de Montréal Montreal, QC Canada; 5 School of Rehabilitation Faculty of Medicine Université de Montréal Montreal, QC Canada

**Keywords:** mental disorders, social stigma, video games

## Abstract

**Background:**

Video game playing is a daily activity for many youths that replaces other media forms (eg, television); it serves as an important source of knowledge and can potentially impact their attitudes and behaviors. Researchers are, thus, concerned with the impact of video gaming on youth (eg, for promoting prosocial or antisocial behavior). Studies have also begun to explore players’ experience of gameplay and video game messages about violence, sexism, and racism; however, little is known about the impact of commercial video games in the sharing and shaping of knowledge, and messages about mental illness.

**Objective:**

The aim of this review was to identify how mental illness, especially psychosis, is portrayed in commercial video games.

**Methods:**

We performed keyword searches on games made available between January 2016 and June 2017 on Steam (a popular personal computer gaming platform). A total of 789 games were identified and reviewed to assess whether their game content was related to mental illness. At the end of the screening phase, a total of 100 games were retained.

**Results:**

We used a game elements framework (characters, game environment/atmosphere, goals, etc) to describe and unpack messages about mental health and illness in video games. The majority of the games we reviewed (97%, 97/100) portrayed mental illness in negative, misleading, and problematic ways (associating it with violence, fear, insanity, hopelessness, etc). Furthermore, some games portrayed mental illness as manifestations or consequences of supernatural phenomena or paranormal experiences. Mental illness was associated with mystery, the unpredictable, and as an obscure illness; its treatment was also associated with uncertainties, as game characters with mental illness had to undergo experimental treatment to get better. Unfortunately, little or no hope for recovery was present in the identified video games, where mental illness was often presented as an ongoing struggle and an endless battle with the mind and oneself.

**Conclusions:**

The game elements of the identified commercial video games included mental illness, about which many perpetuated well-known stereotypes and prejudices. We discuss the key findings in relation to current evidence on the impact of media portrayals of mental illness and stigma. Furthermore, we reflect on the ability of serious video games to promote alternative messages about mental illness and clinical practices. Future research is needed to investigate the impact that such messages have on players and to explore the role that video games can play in fostering alternative messages to reduce the stigma associated with mental illness.

## Introduction

### Background

More than 1.8 billion people play video games worldwide [1]. In a US national survey, 1102 teens aged 12 to 17 years described video gaming as a daily activity [2]. Contrary to a popular belief that views video game playing as a solitary activity, most youth (76%) played video games with others in person or on the Web, and only 24% played alone. In addition to console games (Xbox, PlayStation, or Wii), youth reported playing games on their desktop or laptop computers. First-person shooters, action, and sport games were the top types of video games played; however, most youth did not limit themselves to just one game genre [2].

Given the popularity of video games among young people, researchers have been studying the role that this technology plays in promoting prosocial and antisocial behavior among players. The vast majority of research has focused on the negative impact of video games, describing their potential to lead to aggression, addiction, and depression [3,4]. However, new emerging research challenges this body of work. Notably, Zendle critically analyzed the relationship between violent content in video games and aggressive behaviors [5]. Zendle found that it was players’ frustration with game complexity and difficulty that led them to behave more aggressively after playing rather than the violent content within games.

Emerging literature has also identified the benefits of playing video games in promoting better attention, memory and problem-solving skills, and enhancing one’s ability to cope with failures, manage emotions, and socialize [6]. Furthermore, novel research has focused on the impact of commercial games as health interventions, psychotherapy tools for assessment, and in teaching social skills [7]. In addition, a few reviews have been published to evaluate the effectiveness of video game technology in the treatment of different mental health conditions (eg, depression and anxiety) [8-10].

Studies have also begun to explore players’ experience of gameplay and video game messages about violence, sexism, and racism [11-14]. However, little attention has been devoted to messages about the experience of mental illness in commercial video games [15-17], the impact they may have on attitudes toward and understandings of mental illness, and how they could perpetuate stereotypes and/or stigma about mental illness. Shapiro and Rotter [16] reviewed 96 popular commercial video game characters (highest-selling video games from 2011 to 2013) comparing them with mental illness stereotypes identified in movies [16,18]. Almost all identified video games (93 of 96) depicted at least one character with mental illness while linking mental illness to dangerous and violent behaviors.

Apart from a few published works that looked at mental illness messages in video games [15-17], most of our current knowledge about media portrayals about mental illness concern movies, television (TV), and cartoons. Since the 1990s, researchers have studied the impact of media (eg, TV, newspapers, and cartoons) portrayals of mental illness on the public [19-22] and most recently on children [23]. Specifically, these depictions perpetuated the stereotype that people suffering emotionally might be *mentally ill*, dangerous, or violent [18,24,25]. The portrayal of persons with mental illness as dangerous, incurable, and incapable of controlling their illness has been shown to promote negative attitudes about mental illness and problematic behaviors toward persons with mental illness [26]. In their study on verbalizations (labeling) about mental illness in Disney animated films, Lawson and Fouts [23] found that of the 40 full-length animated feature films produced between 1937 and 2001, 34 films depicted mental illness using terms such as *crazy* or *nuts* *.* Specifically, these terms were used to marginalize and denigrate the characters to whom they referred. Under the influence of media messages, children and youth can begin stigmatizing mental illnesses from a relatively early age.

As described, negative portrayals of mental illness in traditional media are a matter of social concern; however, the impact of mental illness–related content in video games is unknown. To unpack how reality is presented via video games and the potential that these presentations have for shaping youth perceptions, it is necessary to understand the *narrative rhetoric* of game content [27], and the messages that games portray.

### Review Aim

The aim of this exploratory study was to examine the labels used and overall messages about mental illness, especially psychosis, portrayed in video games currently on the market. We explored not only how the experience of mental illness was portrayed in these games but also how treatment and the settings of care were described and represented. We focused on psychosis because it is among the most stigmatized mental illnesses in TV shows and movies, mainly because its unique manifestations through hallucinations and delusions capture the imagination of writers, producers, and audiences [28,29]. Our specific research question was as follows: *How is mental illness, especially psychosis, as well as its context (eg, treatment and settings of care) portrayed in commercial video games?*

## Methods

### Epistemic Framework: Discourses, Messages, and Mental Health Stigma

Informed by critical discourse analysis [30], this work explores the language used to describe and explain mental illness in recently released commercial video games. Critical discourse analysis examines the form, structure, and content of discourse, including the wording chosen and used in a text, to unpack direct and indirect messages sent to the audience.

Theoretically, stigma about mental illness is generated and shaped by labeling and stereotyping. Labeling refers to the use of a term to define a person that impacts people’s attitudes, beliefs about, and behaviors toward that person [31]. Labels ascribed to a person with a mental illness can generate positive or negative connotations about that individual. For example, describing a person with psychosis using words such as *dangerous* can denigrate the person and the person’s illness experience. Stereotyping is the application of a belief about a person that can shape one’s behavior toward that person. Stereotypes can arise from labeling, cultural beliefs, and direct or indirect exposure to a person or a group. If, for example, a person with a history of mental illness commits a crime, especially a violent one, media reports may use terms such as *psychotic* or *out of control* to describe the perpetrator. In such a scenario, a connection is made among the person in question, the person’s mental illness, the labels used, and the crime committed, which perpetuates the stereotype that people with mental illness are violent and dangerous. A review of epidemiological studies shows that the majority of people with mental disorders do not engage in violence against others and that they are actually more at risk to harm themselves than others [32].

Mental illness stigma can lead to 2 problematic behaviors: marginalization, or the separation between *us* and *them* [33], and discrimination, or behaviors that deny social participation or access to resources. Research has shown that stereotypical beliefs about mental illnesses and persons with mental illnesses contribute to social discrimination, including in the domains of jobs and housing [34-36]. Furthermore, it has been suggested that adults’ stereotypical beliefs about mental illness may have originally been acquired through media exposure in childhood and teen years [37-39].

### Search Strategy

On Steam, a popular personal computer (PC) gaming platform, we performed keyword searches using the following terms: “asylum,” “insane,” “crazy,” “mad,” “madness,” “mental,” “psycho,” “psychotic,” “psychosis,” and “schizophrenia” on games released between January 2016 and June 2017, to capture 1.5 years of commercial video game production and dissemination. We selected the Steam platform because it has access to a wide variety of gaming genres and indie games, with PC game sales on this platform being almost as high as all console game sales [40]. In addition to popular and well-known terms or labels associated with psychosis such as “crazy” and “insane,” we searched for formal/medical terms such as “schizophrenia” and “psychosis.” A similar key-terms strategy, a mix of clinical terms and common mental illness labels, was also used in the study by Shapiro and Rotter [16].

A total of 2 reviewers (MF and SM) systematically used the selected keywords on Steam to identify games for inclusion in the analysis. Games were included for full-text review if (1) they were published on Steam between January 2016 and June 2017 and (2) regardless of genre, they featured and/or presented mental illness and/or mental health content. MF and SM first performed a preliminary screen to identify which games were available within the aforementioned period, which yielded a total of 789 games. Furthermore, the reviewers systematically reviewed the Steam game descriptions to exclude games in whose synopses the identified keywords appeared, but not in the context of mental illness (eg, only in the title as in the game *Crazy Fishing*). If a game did meet the criteria, its game information was extracted from Steam’s website and imported into an electronic database for analysis. On the basis of this search criteria, 100 games were retained for data analysis.

### Data Extraction and Coding

We created an electronic database of all retained games. Metadata such as game title, year, genre, and synopsis were entered in an Excel file. The Excel file was then exported into Atlas.ti version 8 (Atlas) [41] to perform thematic analysis. We followed Braun and Clarke’s [42] steps for thematic analysis, informed by an inductive process that began by generating initial codes and themes, and then creating a coding manual. According to the authors [42], thematic analysis is a flexible analytic method that can be effectively applied across a range of epistemological approaches, including discourse analysis [43]. Codes and themes were reviewed and redefined by exploring relationships between code-level and theme-level preliminary analysis. We interpreted the meaning of each identified theme in relation to our research question: *How is mental illness, especially psychosis, as well as its context (eg, treatment and settings of care) portrayed in commercial video games?* Finally, we further unpacked and finalized the analysis by comparing the identified themes with game elements such as genre, game characters, story, atmosphere, game goals, and the game environment, and we used this framework to report our findings.

## Results

### Video Game Taxonomy and General Overview

**Figure 1:**
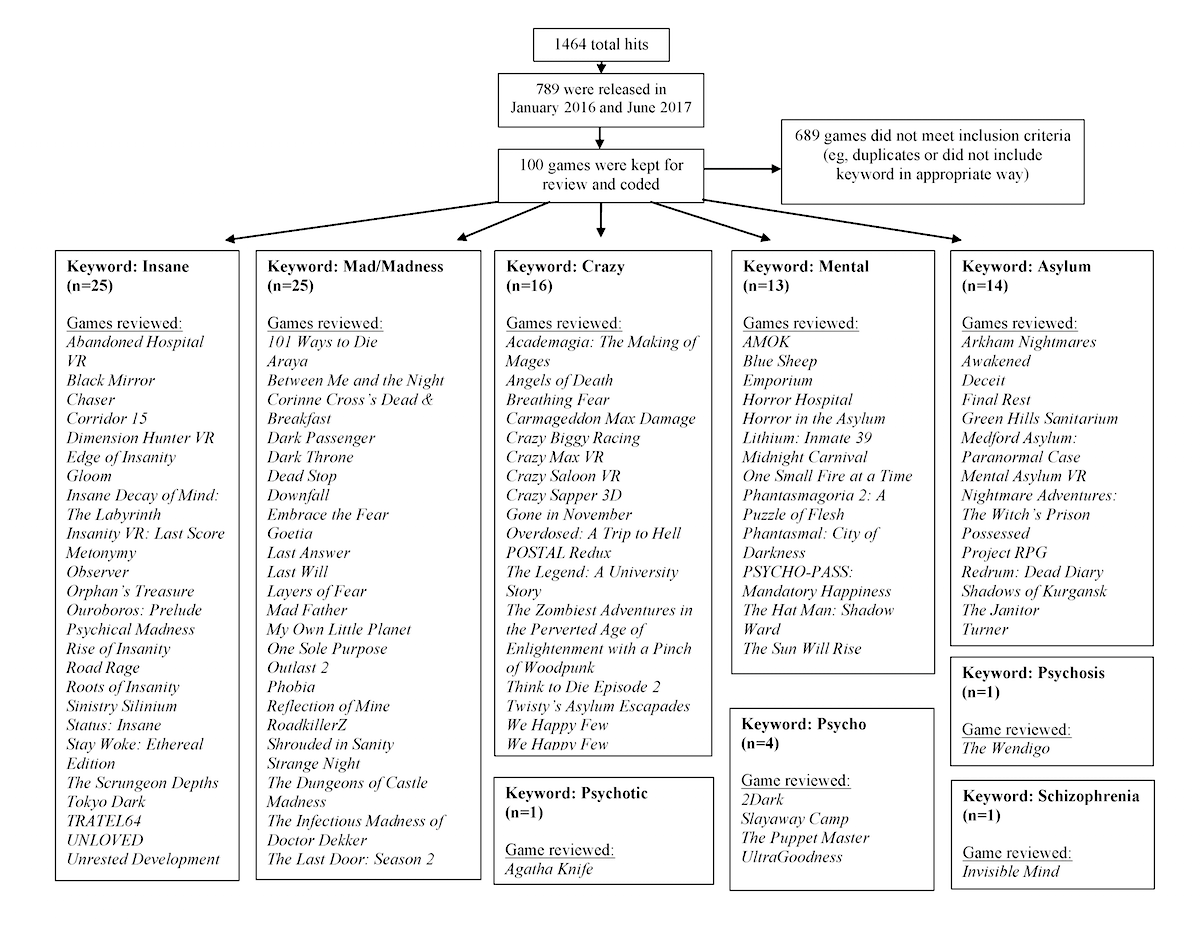
Video games extraction summary.

Information about the 100 games retained for analysis is presented in Table 1 and Figure 1. Overall, the keywords that produced the most hits were “insane” (198/789), “crazy” (244/789), and “mad”/“madness” (244/789), which may reflect the colloquial use of these words to describe excitement, fun, or a “wild” experience. For instance, “crazy” was mainly associated with puzzle games for children and younger audiences. Words such as “crazy,” “insane,” and “madness” were used to convey characters who do not abide by rules or environments that are chaotic or creative. For example, *Crazy Saloon VR* (Monsieur K, 2016) is described as follows:

Discover the Old West with this quirky saloon simulator...There is a free mode where you’ll be able to roam freely around the saloon shooting things and stuff, fully express your craziness creativity!

The game *TRATEL64* (UWILMOD, 2017) explicitly equated “insane” with “eccentric”:

Slice, Snipe, Stab, and Shoot your way through 64 insane targets! Experience the insane (or eccentric if you prefer) personalities of a truly twisted world!

**Table 1:** Game analytics summary for 100 games.

Keyword^a^	Games per keyword, n	Total number of gamer reviews posted on steam		Average cost of games ($CAD), mean	Games with viewing restrictions (age restriction or “not safe for work”), n (%)
		N	Median		
Insane	25	4221	44	9.81	11 (44)
Mad/Madness	25	18,702	78	10.01	5 (20)
Crazy	16	7139	80	12.38	4 (25)
Asylum	14	19,088	20.5	5.42	7 (50)
Mental	13	3704	58	8.84	3 (23)
Psycho	4	621	89	10.29	0 (0)
Psychosis	1	16	—^b^	5.49	1 (100)
Psychotic	1	88	—	12.99	0 (0)
Schizophrenia	1	23	—	0.60	0 (0)

^a^Information accurate as of September 2018 (see Multimedia Appendix 1 for more details).

^b^Not applicable.

Similarly, the description of *Crazy Sapper 3D* (Aratog LLC, 2016) noted the following:

A mad retired General named Boris has hidden a devastating acid vaccine on a secret base...There’s only one man standing in the way of Boris’ dream of creating a super-bomb and enslaving the entire world: a crazy sapper named Max!

In this example, Boris, the antagonist, and Max, the protagonist, both embody being “mad” and “crazy”—stereotypical terms associated with mental illness. The frequency of their occurrence and their use as selling points in the game synopses suggest that terms such as “crazy” and “mad” were accorded (by game creators) or had come to acquire (among gamers) some desirable connotations.

On the other hand, keywords that were more medical in nature produced fewer hits, with “psychotic” resulting in only 4 hits out of 789, “psychosis” in only 3 hits, and “schizophrenia” in only 1 hit. Unlike the word “crazy,” medical terms such as “psychosis” and “schizophrenia” can bring a level of formality and seriousness to games that gamers may not seek, leading to their avoidance or exclusion by game marketers [44,45].

Although the style of game play varied, the genres of games were relatively homogeneous, with most games pertaining to action and adventure genres (83%, 83/100), and a majority (68%, 68/100) being tagged by users as containing horror, violence, and gore.

### Mental Illness and Game Elements

Using the game elements framework [46], we unpacked and described messages about mental illness presented in the identified games. Specifically, we explored these messages based on the following structure: themes associated with the main and secondary characters of the game, the game atmosphere (including location, time period, and overall environment), game goals, and how the game described mental illness and its experience. For each section, we provided an estimate of the percent of games that pertain to the themes presented. This estimate accounts for the potential overlap among themes.

#### Mental Illness and Game Characters: Being Violent, Lost, Lonely, or Helpless

Of the 100 games we reviewed, almost all game characters (whether the main character or secondary characters) were described as “psychotic,” “crazy,” “mad,” “eccentric,” “paranoid,” “unusual,” “evil,” and “insane.” In approximately 30% to 35% of games, characters were portrayed as either *being violent* and/or as *being lost, lonely, or helpless*.

In the reviewed synopses, in approximately 16% of games, the characters were described as *violent or aggressive*. This was especially true of characters who were portrayed as having psychosis, whether explicitly or implicitly, through behaviors such as hearing voices or seeing visions. For instance, violence and “being psychotic” were paired and emphasized in *UltraGoodness* (Rasul Mono, 2017; 80% of 89 gamer reviews were positive), a game that requires players to fight “a fricking army of psychos” and “splatter blood all over the levels.” Although apparently comedic and satirical, the game creates a connection between violence and persons with mental illness (who, in this case, are victims). A similar connection was observed in *Slayaway Camp* (Blue Wizard Digital, 2016; 96% of 409 gamer reviews were positive), whose description reads:

Slayaway Camp is a killer puzzle where you control Skullface, a psychotic slasher bent on slaughtering camp counsellors...this adorable murderer slides around isometric puzzle levels decapitating, squashing, and perforating his bloody victims.

In approximately 15% of games’ synopses, protagonists were described as *lost, lonely, or helpless* in some way. Often, protagonists had to find characters who were lost or were themselves lost. In some games, the journey of mental illness and recovery was therefore represented through a character finding what was lost and overcoming challenges that drive them to “insanity.” For instance, players of *My Own Little Planet* (Lucas Parise, 2017; 63% of 11 gamer reviews were positive) must help a “lost boy who is trapped on a dark and strange world.” They have to “fight [their] deepest fears” to “put an end to [the] infinite loop of madness and fright.”

This theme of finding what was lost was also emphasized in *Tokyo Dark* (Cherrymochi, 2017; 91% of 373 gamer reviews were positive), wherein the protagonist, Detective Ito, in her quest to find her missing partner, begins to “question her own sanity.”

#### Mental Illness and Atmosphere

In unpacking relationships between depictions of mental illness and game atmosphere, approximately 75% to 80% of games had game settings and ambiances that pertained to themes of mental illness.

##### Game Setting

Overall, it was interesting to notice how some games (10%) depicted time periods reminiscent of the Victorian and Edwardian eras, referring to mental health care in historic terms (eg, describing mental health institutions as asylums or sanitoriums). For instance, *Arkham Nightmares* (Tim Rachor, 2016; 20% of 10 gamer reviews were positive) takes place in the “Lovecraftian Arkham Sanitorium” and *Layers of Fear* (Blooper Team SA, 2016; 91% of 6831 gamer reviews were positive) takes place in a “Victorian-era mansion.”

The troubling and ethically questionable history of the treatment of mental illness provided a suitably macabre context for horror games such as *The Hat Man: Shadow Ward* (Game Mechanics, 2016; 67% of 2937 gamer reviews were positive), which tells “the real-life story of the events that took place at the Canton State Insane Asylum fifty years ago, as reported by those who survived.” Its players are “thrust into a living nightmare” in which “electricity and communication with the outside world has been cut off, and the asylum is being terrorized by supernatural beings.” Obviously, supernatural beings did not roam the halls of the Canton State Insane Asylum, but the asylum did exist and had a problematic history. Using it as a setting served to enhance the believability of the game’s story.

Furthermore, mental health care in futuristic games (6%) also described the health care setting in negative ways. In these dystopian futures, the “mentally ill” are restrained, contained, and constantly surveilled. In one such game, *Observer* (Blooper Team SA, 2017; 82% of 1704 gamer reviews were positive), players assume the role of an Observer, “the new front line of neural police” and must “hack into the jagged minds of the insane.” The synopsis further describes the game’s setting as follows:

In this future, anything you think, feel, or remember can be used against you in a court of law...The year is 2084. Those who live have turned to drugs, VR, neural implants- anything to distract themselves from this new reality.

Therefore, in both past and future scenarios (16% of games), individuals with mental illness are depicted as dangerous, in need of containment and separation from society, and requiring treatment that involves constant surveillance by doctors or authority figures, usually in asylums.

Some games (19%) used hospitals and asylums as elements of the environment and story. Asylums and hospitals were described as “abandoned,” “decrepit,” “dark,” or in a “state of disarray.” Asylums were also associated with potentially illegal or strange activity. For example, in *Janitor* (VOS Gaming, 2016; 20% of 25 gamer reviews were positive), the player, Max, is a janitor “sent to an asylum by his boss to repair a broken pipe and a broken electric panel.” The synopsis states the following:

Max knows the asylum is abandoned...His boss warned him not stay long there. Once arrived he quickly notices that something is wrong. Blood everywhere and strange noises can be heard. Short time after looking around a went-wrong experiment of the government rushes to him.

In such games, environments (ie, characters’ homes, cities, or minds) were often described as unsettling, disturbing, and in some form of chaos. For instance, *Insanity VR: Last Score* (Threevol, 2017; 86% of 103 gamer reviews were positive) is set inside the mind of a psychiatric patient:

The game tells the story of a grey future where...it is possible to enter another’s subconsciousness and treat their psychological problems...The player enters the subconscious mind of a former instrument maker. Shortly after the entry...he is locked inside. During the search for a way out, the terrible past of the instrument maker is gradually revealed and the player discovers that he is not alone.

Contrastingly, the game *Horror Hospital* (KNP, 2016; 26% of 156 gamer reviews were positive) takes place in the protagonist’s mind, but the story included the “patient’s recovery and awakening.” In addition, the game setting was also associated with dark, disorganized, supernatural, or disturbing places.

In addition to abandoned, disturbing, and broken spaces, 27% of games were set in dangerous, unpredictable, and violent cities or worlds on the verge of collapse. Houses were portrayed as scary and dark and described as “littered with corpses and other horrors” or “teeming with infernal creatures.” Cities were portrayed as dangerous, “ravaged by war,” “drug-fueled,” and “perilous.” For instance, *Shrouded Sanity* (Steve Gal, 2016; 85% of 221 gamer reviews were positive) is set in an estate filled with “violent inhabitants”:

A strange and repelling madness took hold of the servants...and they will attack on sight...Discover the secrets of the estate, as you look for answers among the violent inhabitants.

A similarly violent environment is depicted in *POSTAL Redux* (Running With Scissors, 2016; 90% of 944 gamer reviews were positive):

Crazed gunmen out for your blood await you around every corner. The only choice is clear: Get them before they get you. Fight back with a devastating arsenal as you make your way through a violence-stricken town.

##### Game Ambiance

Game ambiance refers to the overall “feel” and atmosphere that a video game portrays.

Game synopses (17%) boasted creepy and eerie music and emphasized the supernatural. One game described an asylum as being “full of paranormal activity.” Paranormal activity was a salient and recurring theme, with 11% of games describing buildings or hospitals as being cursed or haunted by ghosts and “otherworldly horrors.” Game environments were described using terms such as “living nightmare” or as spaces where “fear and horror intertwine.”

Creepy and horror-filled atmospheric elements were particularly striking in *Layers of Fear*, a game that requires players to take on the role of an “insane” painter who must navigate a dark, ever-changing mansion to uncover clues about his wife’s death:

Delve deep into the mind of an insane painter and discover the secret of his madness...Uncover the visions, fears and horrors that entwine the painter...A sense of insanity means each turn of the camera may completely change the look of your surroundings.

Creepy imagery was also a key feature of *Medford Asylum: Paranormal Case* (Mzone, 2016; 46% of 43 gamer reviews were positive), where its protagonist “must find what forced workers to stop the renovation. They are frightened by this ‘haunted’ place.”

Horrific, foreboding descriptors were used not only for games’ environments but also for the mental illness itself. For instance, *The Infectious Madness of Doctor Dekker* (D’Avekki Studios Ltd, 2017; 81% of 191 gamer reviews were positive) requires players to explore the “shadow reality” that their “patients inhabit.”

#### Mental Illness and Game Goals

Video game goals refer to the objectives that players must achieve to progress, complete, or win the game. In exploring the relationship between mental illness and game goals, we identified the following themes: solving/uncovering a mystery, survival, finding an escape, and restoring normalcy in approximately 65% to 70% of games.

A large portion of games (30%) dealt with solving puzzles or resolving mysteries. In addition to deploying puzzle solving as a game mechanism, many such games required players to solve murder cases, discover suspects, or piece together “mysterious circumstances.” For instance, the objective of *Rise of Insanity* (Red Limb Studio, 2017; 80% of 287 gamer reviews were positive) is as follows:

As a doctor of psychology, you will discover the gloomiest parts of the human brain. Overcome your fear and find out what really happened to your family...No one knows what has happened to your family...The only suspect is your patient on whom you are testing your experimental treatment methods. Who is responsible for everything that has happened?

The goals of other games involved understanding or discovering phenomena or scenarios that were initially unclear to the games’ characters or players. Examples included game goals such as “discovering the secret to his madness,” “understand what is going on in his mind,” or “discover who is real.” A case in point is *Reflection of Mine* (Redblack Spade, 2017; 85% of 89 gamer reviews were positive) that takes place in the mind of a young girl with a dissociative identity disorder. Its synopsis reads:

The entire game takes place in the broken mind of Lilly Witchgan, and the goal is to discover who is real—Lilly herself or one of her many personalities...Here, madness is represented as it really is—Lilly is a foreigner inher own body...

Other games involved finding what is lost to the character. In one such game, players are tasked with finding the character’s missing client. In another, the protagonist has to search for a friend or family member. However, most games whose plots involved a mysterious element dealt with uncovering or discovering forgotten parts of a character’s past or a traumatic event.

Survival was a significant theme in the games reviewed. Many games (14%) required players to survive passage through dangerous areas, the onslaught of enemies, or even, in the case of one game, “the horrors within.” *Abandoned Hospital VR* (Munsunghyun, 2016; 67% of 3 gamer reviews were positive) requires players to find objects to “stay alive and to escape from the hospital.” Overcoming concrete challenges, obstacles, and traps or some more abstract conceptual tests were also common tropes. In *Between Me and the Night* (RainDance LX, 2016; 68% of 81 gamer reviews were positive), players have to solve puzzles to reveal new places and “overcome the shadows of the night.”

Game goals often (14% of games) involved escaping, either symbolically (eg, “finding an exit to this madness”) or concretely (eg, “escape the asylum”). For instance, in *Awakened* (Jesper Michael Petersen, 2016; 0% of 3 gamer reviews were positive), escaping the “asylum for the criminally insane” involves “escaping the voices in [the protagonist’s] head.” In many games, the escape to avoid being affected was from a hospital or asylum, with some requiring players to flee the “lairs of psychopaths” or the “clutches of an evil doctor.” In *Status: Insane* (Frostbullet, 2017; 95% of 20 gamer reviews were positive), escaping an asylum is the sole objective and story line:

Igor must escape from a mental asylum but it’s not going to be easy. There are two obvious reasons: Igor is constrained by a straitjacket; The asylum’s corridors are filled with armed guards (and other obstacles). In addition to the dangers coming from outside, Igor is going through a war inside his head: His Imaginary Friend is constantly demanding him to cancel his silly escaping attempt.

This game robustly outlines many mental illness stereotypes—that escaping mental illness is futile, that mental health institutions are dangerous, and that patients in these institutions are constrained and confined by force.

Finally, the retention or regaining of sanity was an important goal in the story lines of some games (10%), describing the need to “end the infinite loops of madness and fright” or “maintaining mental health while facing the worst.” For instance, the synopsis of *Phantasmal: City of Darkness* (Eyemobl, 2016; 41% of 115 gamer reviews were positive) stated:

You are fragile. Not just physically, but mentally as well. The grotesque creatures will challenge your very sanity and losing your mind can be a fate worse than death!

Here, the maintenance of sanity is deemed difficult and insanity is deemed the worst consequence. The fragility of sanity was also referenced in *Between Me and the Night*, which boasted an experience that walked players through the “thin path between sanity and madness.”

Recovery and normalcy too were described as fragile states in *Phantasmagoria 2: A Puzzle of Flesh* (Sierra, 2016; 80% of 26 gamer reviews were positive):

[The protagonist] has a steady job...a lovely girlfriend. He’s been out of the mental hospital for exactly one year. All [the protagonist] wants is to live a normal, happy life, but something seems to have other plans...Strange events, inexplicable and terrifying, begin to happen all around him. [The protagonist] begins to doubt his own sanity, and the very fabric of reality.

Video games, thus, commonly depicted mental health as fragile and unpredictable and recovery as difficult to achieve and maintain.

#### The Lived Experience of Mental Illness as a Central Element of the Game

We explored whether and how mental illness was used as a central element in games. Only a minority of games’ descriptions (12%) included more medical terms such as “dissociative identity disorder,” “delusions,” “depression,” and “anxiety.” However, closer examination revealed that even when these terms were used, diagnostic labels seemed to be misapplied or used without a full understanding of their medical meanings. This was especially true for terms associated with psychosis and dissociation. Psychosis and other severe mental illnesses were typically associated with paranoia, lack of control, being followed by a “dark presence,” having an infection, being a “foreigner in her own body,” having a “fragmented sense of self,” being “locked in his own mind,” being “haunted by nightmares,” and/or supernatural and paranormal experiences. For instance, in *The Wendigo* (Warka, 2017; 93% of 16 gamer reviews were positive), players have to participate in a paranormal investigation to uncover the mystery behind an evil spirit that caused psychosis. The game characterizes psychosis as being expressed through violent and unpredictable behavior and the “insatiable desire to eat human flesh.” The association between psychosis and the supernatural is also salient in *Redrum: Dead Diary* (Anarchy Enterprises, 2016; 55% of 27 gamer reviews were positive):

Rose sees dead people who have been killed unjustly, with their mortal wishes left unfulfilled. Her super-practical father refuses to believe her ghostly visions and puts her in an asylum, where she falls into the clutches of the evil Dr. Sigmund Fraud. Help Rose use her psychic powers to solve gruesome murders and outwit a homicidal maniac in this spine-chilling mystery.

Experiences of depression and anxiety were associated with suffering, darkness, and being at the “bottom.” In *Gone In November* (Florastamine, 2016; 53% of 152 gamer reviews were positive), players follow the last days of a man diagnosed with depression, described as follows:

A short experience where your choices and actions don’t matter. Texting messages to a social network account...fencing your apartment to isolate yourself from the outside world - what else someone can possible do when they are at their bottom?

In contrast, the story of *Blue Sheep* (Noetic Games, 2016; 37% of 16 gamer reviews were positive) drew on the real experiences of the game developer:

Highly personal narrative driven by the developers’personal experience with depression and suicide.

Players assume the role of the Outsider, a young black girl, who experiences “the memories of a warrior who once opposed the Beast.” *Blue Sheep* is a puzzle game, in which the players overcome obstacles (a possible metaphor for life’s adversities) to understand the truth behind the Beast, an adversary that symbolizes depression.

*Blue Sheep*, however, was exceptional in its nuanced, realistic portrayal. The majority of identified games (eg, *Reflection of Mine*, *Layers of Fear*, and *The Wendigo*) did not capture the multiplicity of emotions and lived experiences of mental illnesses like psychosis. Instead, they focused on a limited number of negative emotions such as fear and isolation and resorted to dramatizations that left little space for the dignity of those with mental illness.

## Discussion

### Principal Findings

In reviewing the characterization of mental illness in commercial video games, we found that the experience of mental illness, including its treatment and settings of care, was depicted in 100 games. Most of the games we reviewed (97%, 97/100) portrayed and perpetuated well-known stereotypes and prejudices associated with mental illness, namely, that those with mental illness (especially psychosis) are violent, scary, insane, abnormal, incapable, unlikely to get well, isolated, and fearful. Furthermore, some games portrayed mental illness as manifestations or consequences of supernatural phenomena or paranormal experiences. As mental illness was often associated with mystery, being unpredictable, and as an obscure illness, its treatment was also associated with uncertainties, as game characters with mental illness had to undergo “experiment treatment” to get better. Unfortunately, little or no hope for recovery was present in the identified video games, where mental illness was often presented as an ongoing struggle and an endless battle with the characters’ mind and themselves.

Although mental illness can indeed be a scary and isolating experience for some, the stereotypical portrayals of mental illness that we found are problematic because they are partial, negative, and limited [47]. From unvarying characterizations of the sort we found, Adichie [48] said:

The single story creates stereotypes, and the problem with stereotypes is not that they are untrue, but that they are incomplete. They make one story become the only story.

We discuss the key findings further in relation to current evidence on the impact of media portrayals of mental illness and stigma and the ability of video game technology (particularly serious video games) to promote alternative messages around mental illness and clinical practices, and we conclude by presenting future research directions for this field.

### The Power of Media Portrayals and Stigma

As described, the conceptions of mental illness in the games we reviewed tended to reflect common preconceived notions of mental illnesses as dark, scary, isolated, and violent. Furthermore, the person experiencing mental illness was portrayed as having to overcome the negative experience by solitarily winning a war within oneself. The treatment of mental illness was portrayed as an experimental or an uncertain treatment, where psychiatrists were violent, insane, and dangerous. Treatment facilities, mainly represented in the form of asylums, were depicted as abandoned, disturbing, creepy, and haunted. In such settings, patients have to survive or escape to remain alive.

These findings are concerning because the promotion of predominantly negative attitudes and stereotypes is known to foster discrimination against and the marginalization of people with mental illness [49] and treatment settings (eg, mental health hospitals). This is especially salient in the context of video games because of their popularity among youth. Studies have shown that early exposure to characters stereotypically depicted as mentally ill can cause fear and anxiety in young viewers and result in them avoiding individuals with mental illness [22,37-39]. Research has also consistently demonstrated negative consequences associated with adults’ stereotyped beliefs about mental illness [50,51]. Stigma and discrimination also give rise to significant barriers when people with mental illness seek help and access treatment [50,51].

Our findings align with and complement previous research that has examined the impact of TV news on attitudes toward mental illness [25,52,53]. A recent study of a random sample of 400 American news stories about mental illness published from 1995 to 2014 found that the association between mental illness and violence remained strong over time. Although 55% of the analyzed stories linked mental illness with violence (whether interpersonal or self-directed), only 14% reported on recovery from or the successful treatment of mental illness [54]. In a similar vein, research, including 1 experimental study, found that exposure to movies or news stories about mass shootings was associated with harboring negative attitudes toward persons with serious mental illness [25,52,53].

This study’s findings also align with Shapiro and Rotter’s findings by confirming how mental illness is often portrayed through video game characters that are violent, dysfunctional, paranoid, eccentric, afflicted, and broken [16]. Moreover, this study’s findings expand on such analysis by showing that the misrepresentation of mental illness in video games is not limited to video game characters but is fully embedded into the video game experience through its environment, ambiance, and game goals.

### Serious Video Games: The Potential for Alternative Messages and Clinical Practices

#### Co-Design Commercial Video Games for Social Good

Video games have been shown to be effective in promoting learning [55,56] because of their interactivity, repetitiveness, feedback loops, and propensity for emotional learning [57]. As such, they can provide a unique avenue for countering stigmatizing messages and “single story” narratives that create stereotypes. Video games have the potential for telling engaging stories that can shift gamers’ perceptions about mental illness [56,57]. According to Bogost [58], video games have a persuasive power that goes beyond other forms of persuasion, because in video games, learning is mediated by players’ in-game decisions. Bogost argues that the more interactive a game is, the more moved the player will be and, therefore, the greater the potential for intellectual persuasion.

Recently, a few game developers have taken advantage of the interactivity of games to tell engaging, real-life stories about mental illness. For instance, the developers of *Hellblade: Senua* *’s Sacrifice* (Ninja Theory, 2017) consulted with psychiatrists and people living with psychosis to create an in-game experience that depicts psychosis realistically. Similarly, working in partnership with young gamers with lived experience of mental illness and addictions, the developers of *Debris* (Moonray Studios, 2017) also created a gaming experience that focuses on coping with psychosis during a crisis. The game demonstrates how psychosis affects not just the individual but also family members, friends, and fellow citizens [59,60].

Noteworthy is the fact that *Debris* was designed using participatory methodology. Its design process involved the input and perspectives of 5 youth with lived experience of mental illness, Moonray Studio’s founder and project manager, and academic researchers. These stakeholders met for 8 months to discuss video game elements (game characters, story, interactivity, graphics, etc), video game experiences, and messages around psychosis and mental illness [61]. The 5 youth felt that serious video games that attempt to describe the experience of living with mental illness should express empathy, dignity, and compassion. Guided by these values, *Debris* sought to promote messages of compassion and support toward mental illness, rather than fear.

In addition to being developed in consultation with persons with lived experience of and professionals with expertise in mental illness, *Hellblade: Senua* *’s Sacrifice* and *Debris* share certain attributes. Both games acknowledge the link between traumatic experiences and psychosis, demonstrate how illness symptoms (eg, hearing voices) are not always threatening or perceived negatively by persons with psychosis, and depict the different ways in which psychosis can be experienced. They exemplify how video games can employ features, mechanisms, characters, and story lines that can disrupt mental illness stereotypes and potentially contribute significant new understandings of the illness experience.

#### From the Console to the Clinic: Video Games for Clinical Treatment

With growing acknowledgment of their potential for positively impacting learning, video games are now used in treatment settings to promote symptom remission and recovery from mental illness. In a 2012 study, the video game *SPARX* was found to effectively reduce depressive symptoms among adolescents aged 12 to 19 years [62]. The authors concluded that *SPARX* was a potential alternative to usual care for adolescents with depressive symptoms in primary care settings.

Similarly, the game *MindLight* (Playnice Institute) was effective in reducing anxiety symptoms among schoolchildren and children with autism spectrum disorder [63]. The mechanisms deployed in *MindLight* include *exposure techniques* (empirically validated treatment components of cognitive behavioral therapy for anxiety) whereby players are gradually exposed and habituated to threatening cues until they become comfortable with and less anxious about them; *neurofeedback mechanisms* used to promote self-monitoring, relaxation, and concentration; and *attention bias modification* that teaches players to shift attention away from threatening cues and focus on positive aspects of the environment to the end of attaining relevant goals.

Developed for youth and young adults, *Reach Out Central* is a game that enables players to learn skills and information about health-seeking behaviors and progress to higher levels based on the skill and knowledge achieved [64].

Such new-generation, evidence- and lived experience–informed video games can effectively motivate and facilitate mental health learning. They can even open possibilities for envisioning new modalities for providing care to youth and supporting caregivers and health care providers [62-65].

### Limitations

Being based solely on one game platform (Steam) for PC games, our search engine did not capture all available console games (PlayStation, Xbox, etc). Having limited ourselves for feasibility concerns to games published over the course of 18 months, we could not capture the evolution of messages about mental illness in video games over any significant length of time.

Furthermore, in this review, we focused our keywords on psychosis because it is known to be the mental illness that is most frequently and strongly stigmatized [66,67]. Moreover, our list of words was not exhaustive, as words such as “nuts” were not included. Despite not including “depression” as a keyword, we did identify some games with depression. It is possible that some games may not have been captured by the keywords we selected. Finally, not having played the games identified, we could not comprehensively assess how mental illness was depicted in them.

In addition, this review does not fully assess if any of the identified games were troll games or scam games that are posted to generate revenue but do not satisfy game design value or players’ needs. Finally, we recognized that the impact that the 100 reviewed games have on the market is quite heterogeneous. If we look at the game analytics, some games were reviewed by a small number of players, whereas others by more than 15,000 gamers. Game players’ comments can be used to assess the impact of the game; unfortunately, it is a poor measure as not all gamers post a review and gamer satisfaction is based on many different elements (eg, graphics, game experience). Despite this acknowledgment of the variable reach the games reviewed have, this review begins to unpack the negative portrayals of mental illness by game designers and video game industries.

These limitations notwithstanding, we employed novel methodological and analytical approaches to explore mental illness messages in video games. Video games are a new form of media that has yet to be fully examined in the literature base that has hitherto focused primarily on the depiction of mental illness in TV, news reports, and movies. This study’s methods were similar but also different from methods described by Shapiro and Rotter [16]. Our search was comprehensive but also focused, as we looked at all video games on Steam between January 2016 and June 2017, without the use of a predefined analytical framework, as Shapiro and Rotter did. We instead opted for an inductive approach. Finally, we expanded our analysis to assess the representation of mental illness through different game elements. As such, this review contributes significantly to filling an important knowledge gap. Its salience lies in the popularity of video games among young people, and the strong potential for video games in enhancing learning through repetition, interactivity, and immersion.

### Future Directions

Future research initiatives can work to validate the themes identified in this review. An ethnographic study is already underway on the gaming experience of the games identified in this review. Further research exploring players’ perspectives on these games and how they portray mental illness is warranted. Finally, an experimental study is required to assess how and how much the messages about mental illness that this review has identified shape players’ knowledge of, attitudes and behavior toward, and beliefs about mental illness and persons with mental illness.

### Conclusions

The video game industry and its consumers need to be educated about the potential negative impact of ill-conceived messages about mental illness and how these stereotypes can drive discriminatory behavior. As a new generation of collaboratively developed games have shown, much stands to be gained from researchers and clinicians partnering with the gaming industry to create games that can contain and promote positive, nuanced, realistic, and compelling messages about mental illness. Such games can meet gamers’ desires for adventure, pleasure, challenge, and esthetics (eg, *Debris*) as well as meet the video game industry’s desire to produce a successful and popular video game, such as *Hellblade: Senua* *’* *s Sacrifice,* which sold more than 1 million copies within a few months and had 91% of 17,032 gamers rating the game as “Very Positive” on Steam.

## Appendix

Multimedia Appendix 1 Supplementary table of game analytics.

## Abbreviations

CIHR: Canadian Institutes of Health Research
FRQS: Fonds de Recherche du Québec-Santé
PC: personal computer
TV: television
