# Supplementary material for: Gaming With Stigma: Analysis of Messages About Mental Illnesses in Video Games
Source: JMIR Ment Health. 2019 May 8;6(5):e12418. doi: 10.2196/12418 (PMC6707601; doi:10.2196/12418)
Supplement: Multimedia Appendix 1 [file mental_v6i4e12418_app1.pdf]

Game title	Total number of gamer reviews posted on Steam	Percent of gamer reviews that are positive	Cost of game in CAD ($)	Viewing restriction (age restriction or "not safe for work" restriction)
**Keyword: Insane (n=25)**				
Abandoned Hospital VR	3	67%	6.49	No
Black Mirror	104	41%	32.99	Yes
Chaser	600	76%	5.49	No
Corridor 15	2	100%	5.69	No
Dimension Hunter VR	60	76%	6.69	Yes
Edge of Insanity	NA	NA	NA	No
Gloom	16	62%	10.99	No
Insane Decay of Mind: The Labrynth	28	39%	3.29	No
Insanity VR: Last Score	103	86%	Free	Yes
Metonymy	44	50%	Free	Yes
Observer	1704	82%	32.99	No
Orphan's Treasure	NA	NA	NA	NA
Ouroboros: Prelude	6	50%	8.79	Yes
Psychical Madness	12	50%	4.49	No
Rise of Insanity	287	80%	11.49	No
Road Rage	12	16%	39.99	No
Roots of Insanity	184	72%	0.77	Yes
Sinistry Silinium	50	74%	5.49	Yes
Status: Insane	20	95%	5.49	No
Stay Woke: Ethereal Edition	4	75%	2.29	Yes
The Scrungeon Depths	9	89%	8.99	Yes
Tokyo Dark	373	91%	19.99	No
TRATEL64	92	71%	Free	Yes
UNLOVED	500	79%	10.99	Yes
Unrested Development	8	63%	2.29	No

**Average (Range)**	168.84 (2-1704)	144.35 (2 – 1397)	9.81 (Free – 39.99)	11 Yes; 13 No; 1 NA
**Keyword: Mad/Madness (n=25)**				
101 Ways to Die	17	70%	10.99	No
Araya	327	76%	16.99	Yes
Between Me and the Night	89	68%	16.99	No
Corinne Cross's Dead & Breakfast	81	100%	5.49	No
Dark Passenger	10	60%	2.19	Yes
Dark Throne	28	50%	0.59	No
Dead Stop	3	33%	7.99	No
Downfall	405	93%	12.49	No
Embrace the Fear	5	60%	6.49	No
Goetia	162	81%	16.99	No
Last Answer	48	33%	5.49	No
Last Will	64	67%	9.99	No
Layers of Fear	6831	91%	21.99	No
Mad Father	904	96%	5.49	No
My Own Little Planet	11	63%	6.49	No
One Sole Purpose	11	9%	8.79	No
Outlast 2	8834	86%	32.99	Yes
Phobia	11	36%	6.49	Yes
Reflection of Mine	89	85%	8.79	No
RoadkillerZ	13	30%	4.49	No
Shrouded in Sanity	221	85%	5.69	No
Strange Night	78	53%	4.49	No
The Dungeons of Castle Madness	10	80%	10.99	Yes
The Infectious Madness of Doctor Dekker	191	81%	9.99	No
The Last Door: Season 2	259	95%	10.99	No
**Average (Range)**	748.08 (3 – 8834)	656.12 (1 – 7597)	10.01	5 Yes; 20 No
**Keyword: Crazy (n=16)**				
Academagia: The Making of Mages	131	72%	17.99	No
Angels of Death	1458	94%	10.99	No
Breathing Fear	110	83%	10.99	No

Carmageddon Max Damage	1132	76%	18.99	No
Crazy Buggy Racing	10	20%	5.49	No
Crazy Max VR	10	20%	5.49	Yes
Crazy Saloon VR	11	72%	10.99	No
Crazy Sapper 3D	4	50%	5.49	No
Gone in November	152	53%	1.09	Yes
POSTAL Redux	944	90%	9.99	Yes
Overdosed: A Trip to Hell	12	58%	7.79	No
The Legend: A University Story	11	64%	Free	No
The Zombiest Adventures in the Perverted Age of Enlightenment with a Pinch of Woodpunk	NA	NA	NA	NA
Think to Die Episode 2	80	75%	Free	No
Twisty's Asylum Escapades	25	40%	5.49	Yes
We Happy Few	3049	70%	74.99	No
**Average (Range)**	475.93 (4 – 3049)	371.80 (2 – 2134)	12.38 (Free – 74.99)	4 Yes; 11 No; 1 NA
**Keyword: Mental (n=13)**				
AMOK	119	37%	1.09	No
Blue Sheep	16	37%	2.19	No
Emporium	102	71%	2.19	Yes
Horror Hospital	156	26%	1.19	Yes
Horror in the Asylum	31	29%	5.49	No
Lithium: Inmate 39	21	57%	10.99	Yes
Midnight Carnival	64	26%	0.59	No
One Small Fire at a Time	30	100%	10.99	No
Phantasmagoria 2: A Puzzle of Flesh	26	80%	7.79	No
Phantasmal: City of Darkness	115	41%	16.99	No

PSYCHO-PASS: Mandatory Happiness	29	93%	43.99	No
The Hat Man: Shadow Ward	2937	67%	11.49	No
The Sun Will Rise	58	51%	Free	No
**Average (Range)**	264.57 (16 – 2937)	165.85 (6 – 1968)	8.84 (Free – 43.99)	3 Yes; 10 No
**Keyword: Asylum (n=14)**				
Arkham Nightmares	10	20%	3.29	No
Awakened	3	0%	3.29	Yes
Deceit	18717	73%	5.49	No
Final Rest	11	45%	1.09	Yes
Haunted Halls: Green Hills Sanitarium	4	100%	11.49	No
Medford Asylum: Paranormal Case	43	46%	6.49	No
Mental Asylum VR	26	69%	5.49	Yes
Nightmare Adventures: The Witch's Prison	4	100%	14.38	No
Possessed	12	58%	4.99	Yes
Project RPG	16	31%	1.19	Yes
Redrum: Dead Diary	27	55%	7.79	No
Shadows of Kurgansk	157	64%	6.49	Yes
The Janitor	25	20%	1.09	Yes
Turner	33	93%	3.29	No
**Average (Range)**	1363.43 (3 – 18717)	991.36 (0 – 13663)	5.42	7 Yes; 7 No
**Keyword: Psycho (n=4)**				
2Dark	89	73%	28.99	No
Slayaway Camp	409	96%	9.99	No
The Puppet Master	34	82%	Free	No
UltraGoodness	89	80%	2.19	No
**Average (Range)**	155.25 (34 – 409)	139.25 (28 – 393)	10.29	0 Yes; 4 No
**Keyword: Psychosis (n=1)**				
The Wendigo	16	93%	5.49	Yes
**Keyword: Psychotic (n=1)**				

Agatha Knife	88	94%	12.99	No
**Keyword: Schizophrenia (n=1)**				
Invisible Mind	23	34%	0.60	No
**Total Average Across Key Words (Range)**	558.11 (2 – 18717)	434.68 (0 - 13663)	9.37 (Free – 74.99)	31 Yes; 67 No; 2 NA
